# Tunable surface plasmon resonance frequencies of monodisperse indium tin oxide nanoparticles by controlling composition, size, and morphology

**DOI:** 10.1186/1556-276X-9-547

**Published:** 2014-10-02

**Authors:** Keke Ma, Ning Zhou, Meng Yuan, Dongsheng Li, Deren Yang

**Affiliations:** 1 State Key Laboratory of Silicon Materials and Department of Materials Science and Engineering, Zhejiang University, Hangzhou 310027, People's Republic of China; 2 Cyrus Tang Center for Sensor Materials and Applications, Zhejiang University, Hangzhou 310027, People's Republic of China

**Keywords:** Indium tin oxide nanoparticles, Doping, Surface plasmon resonance, Size

## Abstract

Monodisperse indium tin oxide nanoparticles (ITO NPs) with high crystallinity have been synthesized by the rapid thermal injection method and the seed-mediated growth method. We demonstrate that the surface plasmon resonance (SPR) frequencies of ITO NPs can be manipulated from 1,600 to 1,993 nm in near-infrared band by controlling the composition, size, and morphology. The doping Sn concentration in ITO NPs could be controlled via changing the %Sn in the initial feed from 0% to 30%. The shortest SPR wavelength at 1,600 nm with 10% Sn doping concentration indicates highest free electron carrier concentration in ITO NPs, which has direct relationship with doping Sn^4+^ ions. Furthermore, we demonstrate that the SPR peaks can also be tuned by the size of ITO NPs in the case of uniform doping. Besides, compared with the ITO NPs, single crystalline ITO with nanoflower morphology synthesized through the one-pot method exhibit SPR absorption peak features of red-shifting and broadening.

## Background

Currently, the research in the field of plasmonics has spurred a tremendous amount of interest and found a wide variety of applications, including chemical and biological sensing, fluorescence, waveguide, and novel optical devices [1-5]. However, the conventional plasmonic materials such as gold and silver suffer from high-losses caused by interband transition at optical frequency range. Another drawback of noble metals is that their optical properties cannot be adjusted easily as the carrier density cannot be tuned arbitrarily. In addition, the challenge of compatibility with CMOS technology is mainly caused by diffusion problems between silicon and noble metals which seriously affect the performance of the plasmonic and metamaterial devices [6,7]. All the shortcomings severely restrict the realization of plasmonic devices Therefore, a new plasmonic material which can reduce losses and be easily integrated into silicon devices is essential. The energy gap of semiconductors can prevent the interband transition, making it a good alternative in plasmonics. Furthermore, optical bandgap of semiconductors can be tuned in a wide range via various doping level [8,9].

Recently, Teranishi' group [10] found that indium tin oxide nanoparticles (ITO NPs) had the surface plasmon resonance (SPR) frequencies in the near-infrared region which are similar to those of noble metals. Takafumi Sasaki et al. [11] synthesized ITO NPs through precisely controlling the size and morphology. As a consequence, the ITO NPs are expected to be a better alternative plasmonic material in the near-infrared region as a result of metallic properties and inherent special advantages. Therefore, the fundamental research about the morphology, size, and doping levels of ITO NPs has recently gained great interest [12-15]. However, few studies of the synthesis of ITO NPs with controlled SPR properties have been systematically reported.

In this letter, we report a facile solution-phase synthesis of monodisperse ITO NPs. We have demonstrated that through the adjustment of Sn doping concentration, size and shape in ITO NPs, the SPR absorption peaks can be easily tuned in the near-infrared region.

## Methods

Indium acetate (99.99%), tin acetate (99.99%), isocaprylic acid (IA) and tin(II) 2-ethylhexanoic acid, myristic acid (MA), oleylamine (OLA), octadecylamine (ODA), and octadecene (ODE) were obtained from Sigma-Aldrich (Sigma-Aldrich, St. Louis, MO, USA) and used without further purification. Chloroform, ethanol, and hexane were analytical grade reagents (Shanghai Chemical Reagents Co., Shanghai, China).

### Synthesis of ITO NPs

#### The hot-injection approach

Indium acetate (0.9 mmol), tin (II) acetate (0.1 mmol), and MA (3 mmol) were loaded in a three-neck flask with 15 ml of ODE. In the reaction, we selected ODE as solvent because it has high boiling point and is relatively stable and less toxic under high temperature condition. The solution was stirred under vacuum at 110°C for 1 h and 140°C for 1 h. Then the carboxylate solution was rapidly heated at 290°C under argon atmosphere. Another amine solution which contained ODE (2 ml) and OLA (2 ml) was rapidly injected into the reaction flask. After the injection, the color of the solution changed from light yellow to dark green in several minutes. The reaction mixture was then kept at 280°C for 1 h and 240°C for 1 h. The ITO NPs were collected utilizing standard polar/nonpolar solvent precipitation techniques with high-speed centrifuge. The isopropyl alcohol and alcohol were chosen as polar solvent. The process was repeated three times and the final products, ITO NPs, were dispersed in hexane. The Sn doping concentration in the ITO NPs could be controlled from 0% to 30% by changing the %Sn in the initial feeding. The bigger size ITO NPs were synthesized by simply replacing MA and tin(II) acetate with IA and tin(II) 2-ethylhexanoic acid.

#### The seed-mediated growth method

A typical synthetic procedure is as follows. The ODE solvent solution of indium and tin acetates and MA, added with ITO NPs synthesized by the hot-injection approach, was stirred under vacuum at 110°C for 1 h and 140°C for 1 h. Then the solution was rapidly heated to 280°C under argon atmosphere and was injected with 2 ml OLA using syringe pump at different injection rate (1.3 ml/h to 4 ml/h). The size of ITO NPs depends on the injection rate of OLA via syringe pump. The concentration of the ITO NPs seeds was controlled at 35 mg/ml.

#### The one-pot method

The typical one-pot synthesis of ITO NPs was similar to the hot-injection approach. We replaced OLA with ODA (3 mmol) to a flask while other reaction conditions remained. The acetone and chloroform acted as polar and nonpolar solvent to remove residual ODA.

#### Characterization

X-ray diffraction (XRD): XRD measurement was obtained on an X'pert PRO system (PANalytical, Utrecht, The Netherlands) using a Cu Kα radiation (*λ* = 1.5406 Å) at 40 keV and 40 mA.

Transmission electron microscopy (TEM): TEM and high-resolution TEM (HRTEM) images were taken on a Tecnai G2 F30 S-Twin microscope (Philips, FEI, Eindhoven, The Netherlands) at 300 kV. The samples were prepared by depositing a drop of hexane containing the ITO NPs onto carbon-coated Cu grids.

Ultraviolet–visible near-infrared absorption spectra: The optical properties of ITO NPs were performed using a U-4100 (Hitachi, Chiyoda-ku, Japan). The samples for absorption spectra were diluted with tetrachloroethylene.

X-ray photoelectron spectroscopy: X-ray photoelectron spectroscopy was obtained on a Kratos AXIS Ultra DLD (Kratos Analytical, Shimadzu, Hadano, Japan). The samples were prepared by directly depositing a drop of ITO solution onto silicon substrates and then dried in a vacuum oven.

Inductively coupled plasma atomic emission spectroscopy analysis (ICP-AES): The dry ITO powders were dissolved in concentrated hydrochloric acid (38%) and nitric acid. The metal ions were diluted with distilled water. The elemental analysis were performed using an IRIS Intrepid II XSP ICP-AES equipment (Thermo Fisher Scientific, Waltham, MA, USA).

## Results and discussion

### Crystal structure and composition of ITO NPs

**Figure 1:**
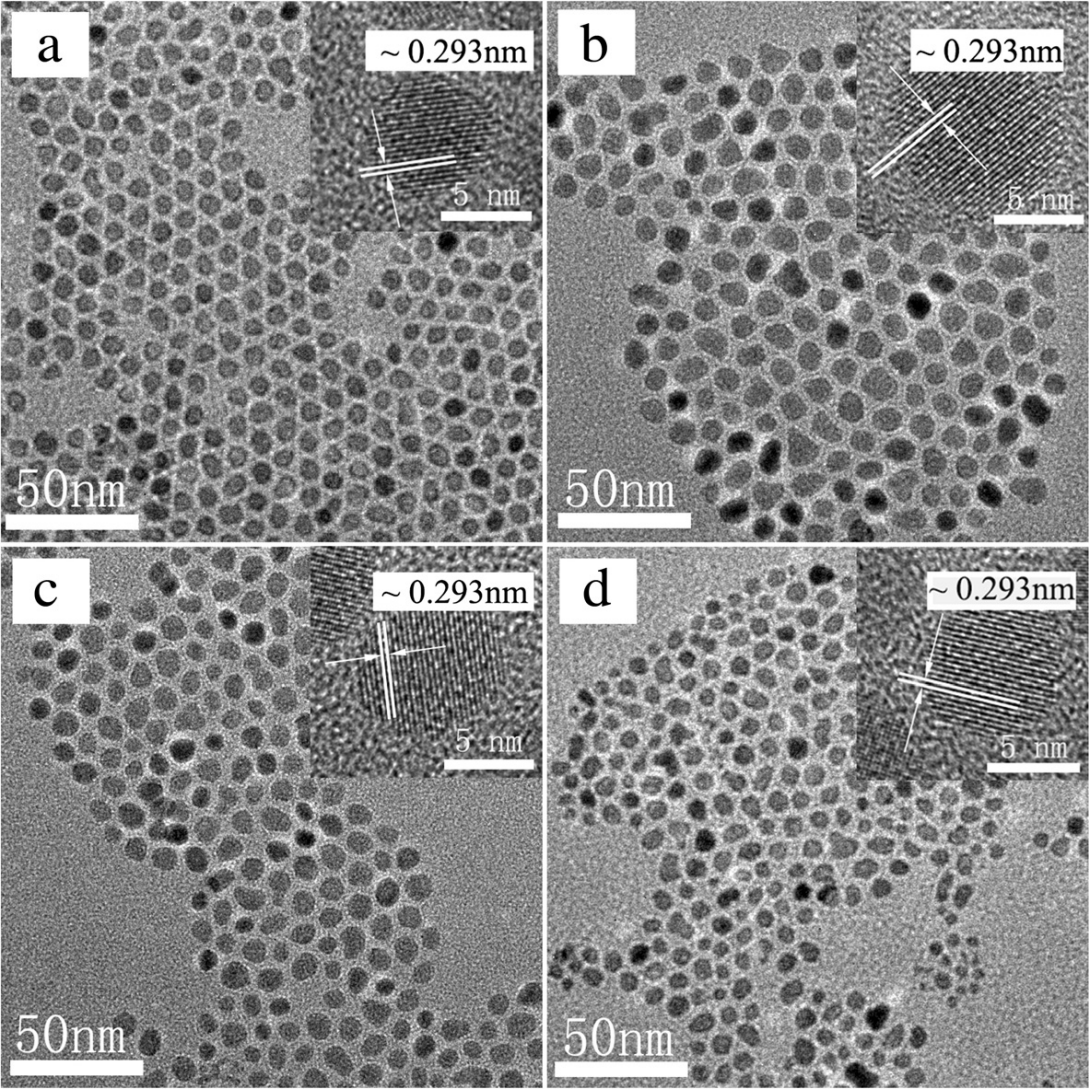
**TEM images of In**_**2**_**O**_**3 **_**and ITO NPs.** TEM images of **(a)** In_2_O_3_ (7.3 ± 1.2 nm, 0% Sn), **(b)** ITO (8.6 ± 1.5 nm, 6% Sn), **(c)** ITO (7.5 ± 1.7 nm, 7.6% Sn), and **(d)** ITO (6.3 ± 1.3 nm, 10% Sn) NPs. All the ITO NPs are synthesized by the hot-injection approach using myristic acid. The insets in all the images show the corresponsive HRTEM images of ITO NPs, in which the lattice spacings of 0.293 nm is consistent with the (222) planes of bulk In_2_O_3._

ITO NPs with different Sn doping concentration were synthesized through changing the concentration of tin precursors in the reagents by the hot-injection approach. The size, shape, and crystallinity of ITO NPs are showed in Figure 1. The different concentrations of metal acetates all succeed in yielding high quality ITO NPs. In the absence of Sn precursors, the sample has well-dispersed In_2_O_3_ NPs which have an average diameter of ca. 7.3 ± 1.2 nm and faceted dots morphology (Figure 1a). In the presence of Sn precursors, the synthesis yields the ITO NPs with average sizes of ca. 8.6 ± 1.5 nm (6% Sn), ca. 7.5 ± 1.7 nm (7.6% Sn), and ca. 6.3 ± 1.3 nm (10% Sn). The single crystalline nature of nanoparticles is confirmed by HRTEM observations (insets in Figure 1, top right). The average lattice spacing is 0.293 nm which corresponds to the (222) planes of bulk In_2_O_3._ The most notable feature of these ITO samples is that the morphology of ITO NPs tends to be more irregular, and the size becomes somewhat smaller when increasing the Sn doping concentration. This phenomenon is consistent with the fact that the emergence of impurity ions can suppress the growth of grain because the mismatch between different atoms can produce lattice distortion.

**Figure 2:**
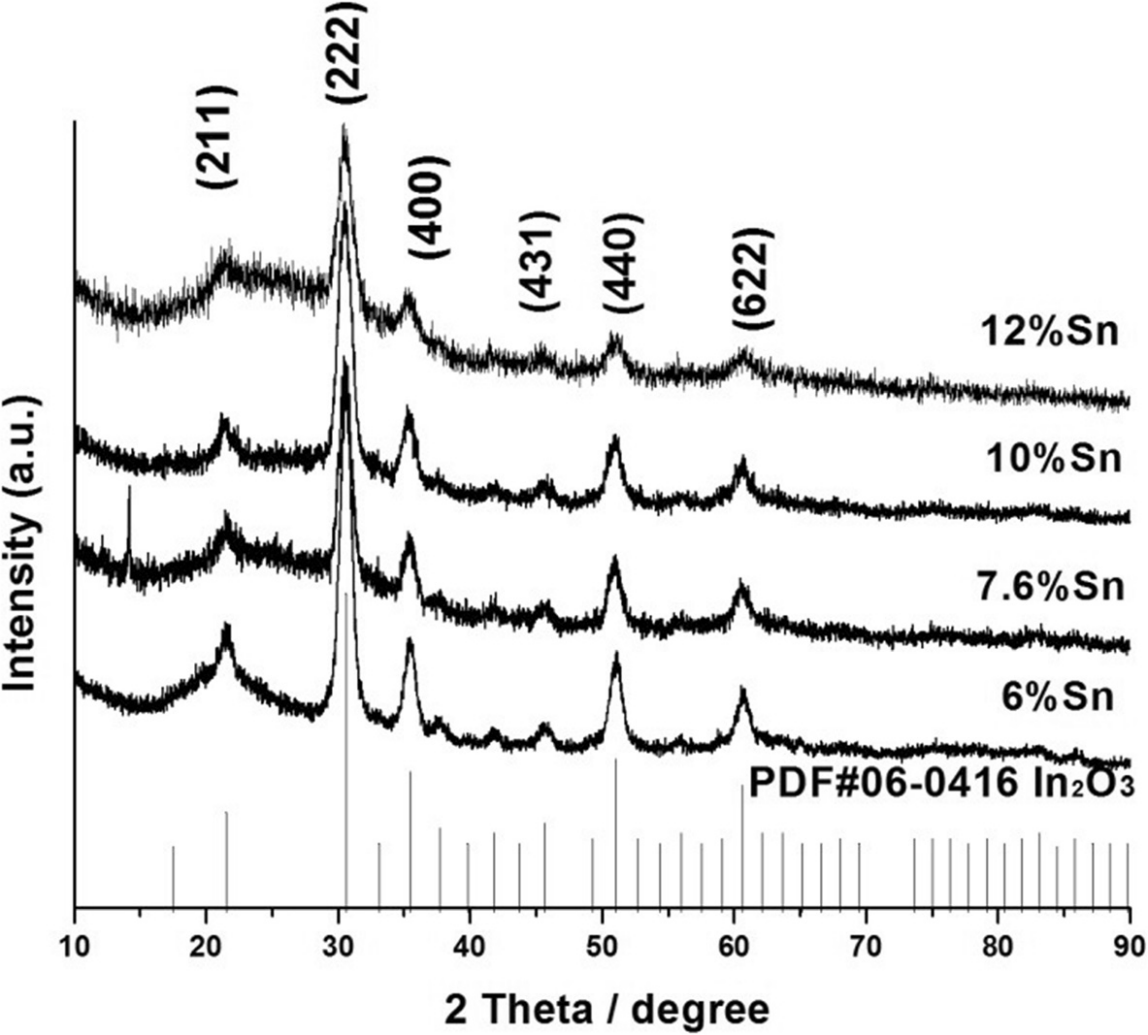
**XRD spectra of ITO NPs prepared with the hot-injection approach.** The initial concentrations of Sn are indicated in the graph. No additional annealing process was performed before analysis.

The XRD patterns of ITO samples prepared with different initial tin concentration are showed in Figure 2. The XRD patterns match very well with the pattern of the cubic bixbyite structure (ICDD Card No. 6-416). As shown in Figure 2, the distinct crystalline peaks found in all the samples mean that all the synthesized NPs crystallize well. The XRD peaks are more broadening as the Sn concentration increases, which is consistent with the fact that the higher concentration of Sn doping causes lattice distortion more seriously. In all these XRD patterns, no SnO_2_ or SnO peaks appear, which means that indium atoms are just replaced with tin atoms.

The ITO NPs assembly samples fabricated by drop-coating were used for the XPS analysis, which can determine the final composition of ITO NPs. Importantly, the binding energy of Sn 3d_5/2_ and Sn 3d_3/2_ at 486.7 and 495.3 eV corresponding to Sn^4+^ bonding state represents that a certain amount of Sn^4+^ ions have substantially replaced In^3+^ ions in the lattice of NPs which directly attribute to the free carriers in the ITO NPs. The element sensitivity factor method [16] is presented in the following equation:

(1) $$\mathrm{Atomic}\;\mathrm{Conc}.\%=\frac{I_{n}}{{\mathrm{RSF}}_{n}}/\sum_{i}\frac{I_{i}}{{\mathrm{RSF}}_{i}}$$

Where I_*i*_ is the integral area of the peaks of the elements, RSF_*i*_ is the relative sensitivity factor for each element. The results of doping concentration of Sn are summarized in Table 1. In addition, the percentage of tin doping achieved ([Sn]/([Sn] + [In]) in each sample was measured by ICP-AES analysis (Table 1). Both analyses reveal that the actual percentage of tin doping in ITO NPs is quite close to the initial Sn concentration. Thus, we can change Sn doping in the ITO NPs effectively by controlling the thermal injection amount of Sn from 0% to 30% in the initial feeding.

**Table 1:** Doping concentration of Sn in ITO NPs measured by XPS spectra and ICP-AES

**Initial Sn concentration (at %) ([Sn]/([Sn] + [In])**	**Sn doping**	**Concentration (at %)**
	**XPS**	**ICP-AES**
6.0	5.8	5.9
7.6	7.3	7.3
10	10.3	9.5
12	11.4	11.5

### Sn doping controlled SPR of ITO NPs

**Figure 3:**
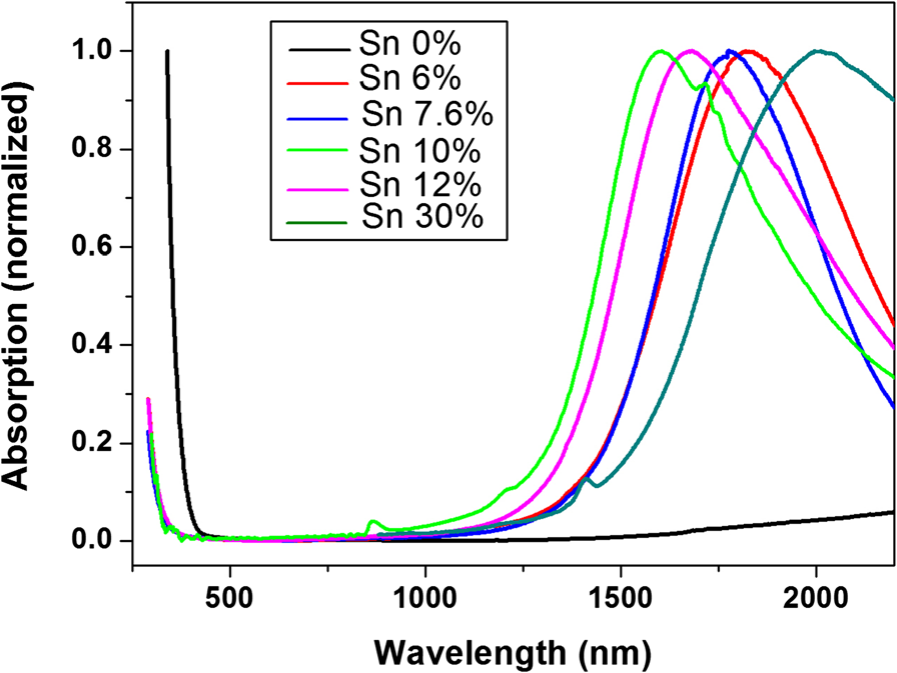
**Normalized UV–vis-NIR spectra of ITO NPs with different doping concentrations of Sn (hot-injection approach).** The SPR peak wavelengths of 6%, 7.6%, 10%, 12%, and 30% Sn-doped ITO NPs are 1,826, 1,774, 1,600, 1,678, and 1,993 nm, respectively.

The ultraviolet–visible near-infrared (UV–vis-NIR) absorption spectra indicated with the Sn doping concentration in ITO NPs is shown in Figure 3. Obviously, there exists no SPR absorption peak in the In_2_O_3_ NPs, while all the ITO samples have SPR peaks in the near infrared region which once again confirms that the doping of Sn in ITO NPs contributes to high free electron concentration. The UV band-gap absorption of all the ITO samples is significantly blue-shifted compared to the undoped In_2_O_3_ sample, which have been described as Burstein-Moss effect in semiconductor materials. As the conduction band is filled with free electron, the band-gap becomes wider as a consequence of combined action between the intrinsic band-gap and carrier concentrations. The direct band-gap is 3.19 eV for the undoped In_2_O_3_ NPs, while it increases to 3.47 eV for the ITO samples. There is about 0.28 eV band gap expansion directly attributed to the free carriers provided through Sn^4+^ ion doping. The existence of a wide band-gap inhibits interband transition nearly. Thus, heavily doped semiconductor with high carrier mobility can act as a low loss plasmonic material.

The SPR peak wavelengths of 6%, 7.6%, 10%, 12%, and 30% Sn-doped ITO NPs are 1,826, 1,774, 1,600, 1,678, and 1,993 nm, respectively. According to the generalized Drude-Lorentz theory, the plasma frequency (*ω*_*p*_) is given as the follow expression [17]:

(2) $$\omega_{p}=\sqrt{\frac{Ne^{2}}{\epsilon_{\mathrm{opt}}\epsilon_{0}m*}}$$

Where *N* is conduction electron density, m* is the effective optical mass of conduction electrons, *ϵ*_0_ is the vacuum permittivity, and *ϵ*_opt_ is the one measured in transparent spectral region which is known from the refractive index of undoped semiconductor. In Equation (2), the plasma frequency has direct relationship with the carrier concentration. The ITO NPs with 10% Sn doping have the shortest SPR wavelength which means that ITO NPs with 10% Sn doping have the highest electron density. Under this condition, whether increasing or decreasing doping concentration will lead to a gradual red shift of the SPR peak, which is consistent with other researches about ITO film that there is a highest free carrier concentration when 10% Sn is doped [18,19]. In the case of heavy Sn doping, partial Sn^4+^ ion is readily reduced to Sn^2+^ for the reason that free electrons are bound around the Sn_In_^*•*^, and the concentration of oxygen vacancy decreases [20]. Thus, the SPR frequency in ITO NPs can be easily controlled by changing the concentration of the Sn doping.

### Size-controlled SPR of ITO NPs

**Figure 4:**
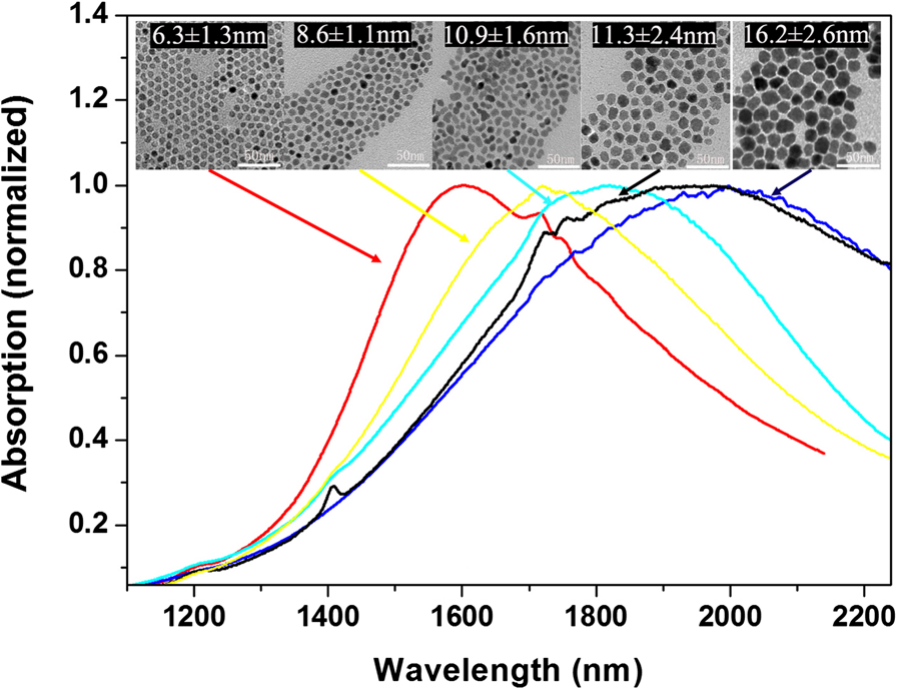
**UV–vis-NIR spectra of different size (insets) ITO NPs with doping 10% Sn.** The SPR peak wavelengths were 1,600, 1,723, 1,799, 1,924 and 1,978 nm.

Similar to metallic plasmonic materials, the SPR absorption peaks of ITO NPs can also be tuned by controlling sizes when the Sn doping concentration was set to 10%. Through tuning the length of carbon chain in fatty acids and the degree of ligand protection, we can synthesize monodisperse ITO NPs with a different size in the hot-injection system. The corresponding TEM images and UV–vis-NIR spectra are presented in Figure 4. The SPR peaks are gradually red shifting from 1,600 to 1,978 nm when the size of ITO NPs increases from 6.3 to 16.2 nm. In order to eliminate the influence of the composition on the SPR peak position, the Sn doping concentration in all the samples was controlled around 10%. By the ICP-AES analysis, the Sn doping concentration of the ITO NPs is 10.1%, 10.8%, 10.0%, and 10.3%, as the size increases gradually, which manifests that we can achieve the same Sn doping concentration ITO NPs with a different size. Thus, the seed-mediated growth method is effective to tune the size of monodisperse ITO NPs. The injection rate of OLA via a syringe pump is 4 ml/h and 1.3 ml/h, corresponding to ITO NPs with a diameter of ca. 8.6 ± 1.1 and 10.9 ± 1.6 nm. Besides, in the hot-injection approach, the ITO NPs have an average diameter of ca. 16.2 ± 2.6 and 11.3 ± 2.4 nm in presence of 500 and 750 μl IA. It was expected to replace acetate in raw materials with myristic or isocaprylic acid when the reaction temperature is higher than the boiling point of acetic acid. Reasonably, as the stabilizing ligands, the length of carbon chain of myristate is longer than isocaprylic acid; primary nanocrystals would be more sterically hindered in the subsequent crystal growth process. In addition, the amount of isocaprylic acid also affects the sizes of ITO NPs.

However, this size dependence of SPR peaks is qualitatively contrary to what is anticipated by the classical Drude model. According to the classical Drude model theory, the enhanced relaxation rate of a spherical particle arises from additional grain-boundary scattering [6] as described in Equation (3):

(3) $$\gamma =\gamma_{0}+A\frac{v_{F}}{R}$$

where *γ*_0_ is the relaxation constant of bulk material, *v*_F_ is the Fermi velocity of electrons, *A* is a dimensionless empirical constant, depending on details of the scattering process, and *R* is the average size of spherical plasmonic particles. In Equation (3), the relaxation rate, depending on the size of the plasmonic particles, is that smaller particles result in a larger *γ* and, hence, higher losses. Furthermore, according to the extended Drude model that accounts for size-dependent surface scattering, the SPR peaks actually undergo a gradual red shifting with decreasing radius for a fixed doping concentration. The Drude model is in contrast to our experimental results, for that to semiconductor nanoparticles; the carrier densities are several orders of magnitude lower than that of metals, and quantum confinement effects are not taken into account. In order to more accurately explain the relationship between the SPR peaks and size, the Lorentz model is needed for small SPR-supporting semiconductors NPs. A similar phenomenon has been recently discovered, and the Lorentz model was used to interpret a blue shift of SPR energies of Ag nanoparticle [21] and photo-doped ZnO nanocrystals [22] with decreasing size.

### Morphology controlled SPR of ITO NPs

**Figure 5:**
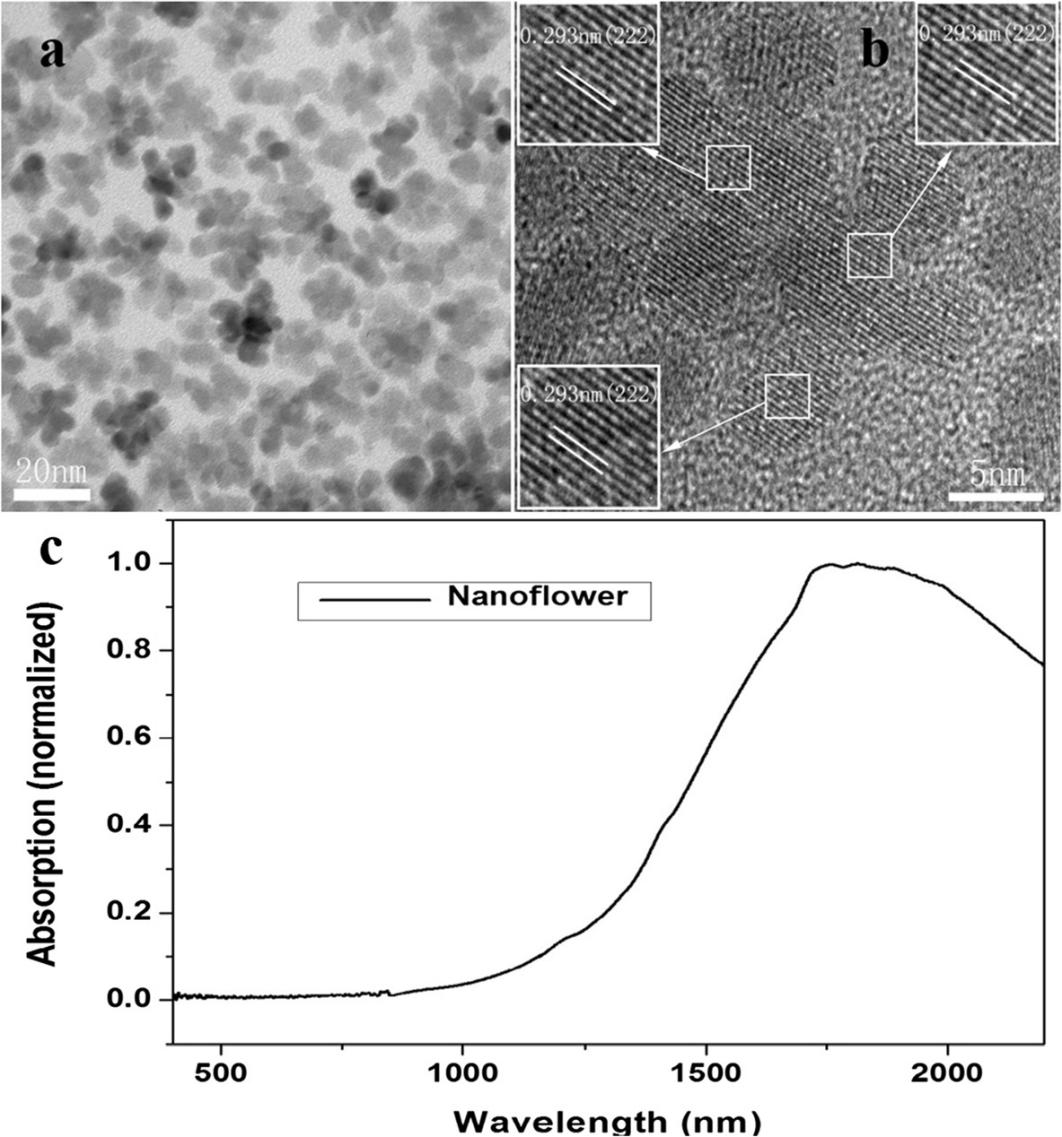
**TEM and HRTEM images and UV–vis-NIR spectra of ITO nanoflowers. (a)** TEM image of ITO nanoflowers and **(b)** HRTEM image of a representative nanoflower. **(c)** UV–vis-NIR spectra of ITO nanoflowers (the one-pot method) with doping 10% Sn.

The morphology of ITO samples synthesized by the one-pot method is distinctive. A typical TEM image of ITO nanoflowers is shown in Figure 5a. The morphology of ITO NPs is identified as flowerlike clusters with an average diameter of ca. 20 nm. The crystalline nature of the nanoflowers is confirmed by the HRTEM image shown in Figure 5b. Importantly, the clear and visible lattice fringes are found to extend across the entire nanoflower structure, and the lattice spacing in each primary particle is visible and has an average size of about 0.293 nm matching (222) d-spacing of bulk In_2_O_3_ (insets of Figure 5b). There is obviously no interface between two single particles, indicating that the nanoflower structures were spontaneously formed by oriented attachment. In the one-pot method, smaller bcc-ITO NPs can assemble into three-dimensional (3D) nanostructures. It is plausible that ITO NPs can lead to 3D-oriented attachment under low ligand concentrations which was described as ‘limited ligand protection’ (LLP) [23,24]. As for the existence of ODA in the initial stages of reaction, it is not sufficient for all MA to replace all acetate when the partial hydrolysis reaction between free carboxylic acid and ODA in solution has occurred prior to the temperature at 280°C. At the early stages of the growth, the ITO NPs' nucleus surface is still covered with a certain amount of acetate anions which reduce the MA surface attachment to some extent and cannot provide enough steric hindrance. From the view of the thermodynamics, 3D-oriented attachment occurs by attractive Van der Waals forces among ITO NPs and will decrease the total surface energy; thus, the surface Gibbs free energy is reduced. The Sn doping concentration in ITO nanoflowers is 9.6% through the ICP-AES test. These 3D nanoflowers have prominent advantages of fairly large surface area which is especially beneficial in the sensing applications.

The UV–vis-NIR absorption spectrum of ITO nanoflowers is shown in Figure 5c. The SPR peak for ITO nanoflowers is around 1,844 nm, a red shift from 1,600 to 1,844 nm compared with ITO nanospheres. Simultaneously, the SPR peak becomes asymmetric and broadening. These phenomena can be interpreted as that the smaller NPs in the ITO nanoflowers have their own SPR phenomenon, which leads to the existence of multiple surface plasmon resonance in complex nanoflowers. Once the mutual coupling effect occurs among the multiple SPR modes, it can lead to SPR absorption peak red-shifting and broadening [25,26].

## Conclusions

In summary, we have employed ITO NPs and their SPR property as our targets to investigate the synthesis of composition, size, and shape-controlled ITO NPs. Through the hot-injection approach, we successfully obtained the monodisperse colloidal ITO NPs with the SPR peaks that can be tuned from 1,600 to 1,993 nm in near-infrared band by changing the %Sn in the initial feed from 0% to 30%. Through the changing activity in the reaction precursors and seed-mediated growth method, we synthesize different sizes of ITO NPs. As the size gradually increases, the SPR peaks can be controlled from 1,600 to 1,978 nm. We also found that in the one-pot method, LLP leads to nanoflower structure formation with SPR absorption peak red-shifting and broadening. Finally, our study focuses on the SPR property of ITO NPs affected by its composition, size, and morphology as a better alternative plasmonic material in the near-infrared region.

## Abbreviations

IA: Isocaprylic acid; ICP-AES: Inductively coupled plasma atomic emission spectroscopy analysis; ITO NPs: Indium tin oxide nanoparticles; MA: Myristic acid; ODA: Octadecylamine; ODE: Octadecene; OLA: Oleylamine; SPR: Surface plasmon resonance; TEM: Transmission electron microscopy; XPS: X-ray photoelectron spectroscopy; XRD: X-ray diffraction.

## Competing interests

The authors declare that they have no competing interests.

## Authors' contributions

KM performed the experiments, collected and analyzed the data, and wrote the paper. NZ, MY, and DY helped with the data analysis and wrote the paper. DL conceived the experiments, analyzed the results, and wrote the paper. All authors read and approved the final manuscript.
